# Editorial: Inborn errors of carbohydrate metabolism

**DOI:** 10.3389/fgene.2024.1430414

**Published:** 2024-06-20

**Authors:** Iván Martínez-Duncker, Ida Vanessa Doederlein-Schwartz, Melania Abreu-González, José Elías García-Ortiz

**Affiliations:** ^1^ Laboratorio de Glicobiología Humana y Diagnóstico Molecular, Centro de Investigación en Dinámica Celular, Instituto de Investigación en Ciencias Básicas y Aplicadas, Universidad Autónoma del Estado de Morelos, Cuernavaca, Mexico; ^2^ Department of genetics, Universidade Federal do Rio Grande do Sul (UFRGS), Porto Alegre, Brazil; ^3^ Genos Médica Centro Especializado en Genética, México. Centro Médico ABC, Mexico City, Mexico; ^4^ División de Genética, Centro de Investigación Biomédica de Occidente, Centro Médico Nacional de Occidente-Instituto Mexicano del Seguro Social (CMNO-IMSS), Guadalajara, Mexico

**Keywords:** CDG (congenital disorder of glycosylation), lysosomal storage disease, Pompe (glycogen storage disease type 2 / II), diabetes mellitus, rare disease (RD), ERT (enzyme replacement therapy), adult polyglucosan body disease (APBD), metabolism

The Inborn Errors of Carbohydrate Metabolism Research Topic offers a view on the ever-evolving field of metabolic disorders, particularly on a heterogeneous subgroup of inborn errors that are caused by pathogenic variants in human genes coding for proteins involved in catabolic and anabolic pathways of carbohydrates (Witters et al., 2021). The Research Topic includes original research articles, case reports, systematic reviews and mini-reviews, that highlight unique case reports, recent advances, innovative diagnostic techniques, and emerging therapies that are shaping the landscape of treatment. Significant challenges are addressed in terms of diagnosis, management, and therapeutic intervention of congenital disorders of glycosylation (CDGs), glucose/galactose malabsorption disorder, lysosomal storage diseases (LSDs), adult polyglucosan body disease (APBD) and diabetes mellitus type 1.

CDGs are a group of inherited metabolic diseases that disrupt glycan synthesis and thus glycoprotein and glycolipid synthesis, including glycophosphatidylinositol (GPI) anchors (Ondruskova et al., 2021). They are usually multisystemic in nature, frequently involving central nervous system disease. In this research topic, four works present molecular and clinical contributions to the comprehension of CDGs, shedding light into autosomal recessive ALG2-CDG, as well as into a small group of CDGs that are X-linked: SSR4-CDG, PIGA-CDG and ATP6AP2-CDG. In a case report and literature review Wang et al. report on the first Chinese male patient affected by an ultra-rare X-linked recessive disorder called SSR4-CDG (OMIM #300934). Whole exome sequencing (WES) revealed a novel *de novo* hemizygous variant c.269G>A (p.Trp90*) in *SSR4* that codes for a protein associated with the translocon TRAP complex, thus affecting endoplasmic reticulum protein transport and glycosylation. The patient presented psychomotor retardation, microcephaly, abnormal facial features, and nystagmus as well as an abnormal carbohydrate deficient transferrin test (CDT) (Bruneel et al., 2020). In a case report, Salinas-Marin et al. present the first Mexican male child affected with PIGA-CDG (OMIM #300868), also an X-linked recessive CDG that disrupts the initial steps of GPI-anchor biosynthesis. A novel *de novo* hemizygous variant *PIGA* c.145G>A (p.Val49Met) was identified by WES and confirmed to be pathogenic through flow cytometry and complementation assays in PIGA knockout cells by measuring GPI-anchored proteins DAF and CD59. The child presented a moderate to severe phenotype characterized by neurological and gastrointestinal symptoms, including megacolon that highlights this case and that possibly results from involvement of GPI-anchored proteins in the development of the enteric nervous system. In an original research article, Fang et al. present the first Chinese male child affected by another ultra-rare X-linked hereditary condition (four patients reported worldwide) known as ATP6AP2-CDG (OMIM #301045) caused by a novel hemizygous mutation in *ATP6AP2* c.185G>A (p.Gly62Glu) identified by WES. This gene codes for ATP6AP2 [ATPase H^+^ transporting accessory protein 2, also known as the (pro) renin receptor] that has a dual function, both interacting with renin or prorenin on the cell surface to affect protein activity and acting as a part of the proton pump associated with adenosine triphosphatase. The patient presented with recurrent jaundice, *cutis laxa*, cirrhosis, growth retardation, coagulopathy, anemia, and cardiomegaly. In contrast with previously reported European patients he did not present immunodeficiency and presented cardiomegaly, expanding the known phenotype of the disease. Interestingly, in a disease model using HEK293T cells, the mutation was shown to dysregulate autophagy and mTOR signaling and metabolites involved in lipid metabolism pathway were found downregulated, noting that this disease causes altered lipid metabolism in patients, particularly cholesterol. In a case report, Martínez-Duncker et al. present the first Mexican child affected by ALG2-CDG (OMIM #607906), presenting as a congenital myasthenic syndrome (Ohno et al., 2023). *ALG2* encodes an α1,3 mannosyltransferase involved in the early steps of *N*-glycosylation. Whole genome sequencing revealed the presence of two *ALG2* variants in compound heterozygosity: a novel variant c.1055_1056delinsTGA p.(Ser352Leufs*3) and a variant of uncertain significance (VUS) c.964C>A p.(Pro322Thr). The patient presented an abnormal CDT test and interestingly, a recently reported ALG2-CDG diagnostic biomarker was found in transferrin and plasma glycoproteins, consisting of a linear heptasaccharide (Sialic acid-Galactose-N-acetylglucoamine-Mannose2-N-acetylglucosamine2), confirming its usefulness in confirming molecular diagnosis. Also included is a comparative phenotype overview of the fourteen patients reported worldwide.

Regarding the catabolic perspective of carbohydrate metabolism, LSDs encompass a group of genetic disorders characterized by lysosomal dysfunction. These disorders arise from deficiencies in specific lysosomal enzymes, membrane transporters, or lysosomal structural proteins, leading to the accumulation of undegraded substrates within lysosomes (Filocamo and Morrone, 2011). The progressive accumulation of these substrates disrupts cellular homeostasis, resulting in cellular dysfunction, tissue damage, and multi-organ pathology. LSDs typically exhibit a wide spectrum of clinical manifestations, including neurological impairment, skeletal abnormalities, hepatosplenomegaly, and cardiorespiratory complications, among others. The onset, severity, and progression of symptoms vary widely among different LSDs, influenced by factors such as the specific enzyme deficiency, the type of accumulated substrate, and the affected tissues and organs. In this Research Topic five contributions present molecular, diagnostic and therapeutic contributions to the comprehension of lysosomal disorders including Gaucher Disease (GD), Mucopolysaccharidosis type II (MPS II) and Pompe Disease (PD).

In an original research article, Zhang et al. describe interesting data in a retrospective study in China on the clinical characteristics, genotypes, and management strategies of 130 patients with MPS II, a rare, progressive, and ultimately fatal X-linked lysosomal storage disorder caused by variants in *IDS* that encodes for the iduronate-2-sulfatase (IDS), an *exo*-sulfatase that hydrolyzes the C2-sulfate ester bond from nonreducing terminal α-L-iduronic acid residues in heparan sulfate and dermatan sulfate (OMIM# 309900). The study analyzed clinical manifestations, auxiliary examination, pathogenic gene variants, enzyme activity, and surgical history. The most common symptoms in the patients were claw-like hands, coarse facial features, birthmarks, delayed development, and inguinal or umbilical hernia. The most common cardiac manifestations were valve abnormalities, with mitral/tricuspid valve regurgitation (71.9%) and aortic/pulmonary valve regurgitation (36.8%). The study found 43 different IDS pathogenic gene variants in 55 patients, including 16 novel variants, concentrated in exon 9 (20% = 11/55), exon 3 (20% = 11/55), and exon 8 (15% = 8/55). The most common and earliest surgery was hernia repair. Three patients died of respiratory failure. The study provides additional information on the clinical, cardiac ultrasound, and surgical procedure in MPS II patients and expands the genotype spectrum of MPS II.

In an original research article, Colburn and Lapidus analyze PD newborn screening data. PD is a rare lysosomal storage disorder, is caused by genetic variants in *GAA* that encodes the acid alpha-glucosidase (GAA), an enzyme that degrades alpha-1,4 and alpha-1,6 linkages in glycogen, maltose, and isomaltose (OMIM #232300). By studying a cohort of 11.6 million newborns, the largest relevant dataset to date, they determine a new prevalence at birth of 1:18,711, with no evidence of difference across European, Latin American, or Asian ancestry. The study compares the results based on direct detection of disease and analyzed using a binomial method along with power analysis with other methods for estimating the frequency of rare genetic diseases. The study also compares these results to previous analyses to offer a framework for evaluating ‘frequency’ data that can be applied to other rare, genetic diseases, along with methods to assess the quality of estimates. The study demonstrates the implications of sample size and frames a discussion on its influence on the reliability of results when extrapolating to a population beyond the study dataset.

The availability of enzyme replacement therapy (ERT) with alglucosidase alfa (human recombinant GAA) has changed the natural history of PD, contributing to a longer survival, longer preserved muscle performance and increased quality of life (Kishnani et al., 2007). A systematic review and meta-analysis of 1,722 articles evaluating ERT for infantile-onset Pompe disease (IOPD) was conducted by Dornelles et al. in Brazil. The results showed that alglucosidase alfa potentially improved left ventricular mass, time to start ventilation (TSV), and survival in IOPD patients over the natural history of PD/placebo. There were no differences between the pre- and post-ERT period for myocardial function and psychomotor development. Adverse events after ERT were mild in most cases. The study concluded that alglucosidase alfa potentially improves LV mass, TSV, and survival in IOPD patients, with no important safety issues. The authors suggest that alglucosidase alfa could be a potential treatment for PD, with no significant safety issues. The study also highlights the need for further research on the safety and efficacy of ERT in IOPD patients.

Additionally, in an original research article, Carter et al. present study findings involving patients with late-onset Pompe disease (LOPD) who switched from alglucosidase alfa to avalglucosidase alfa and that experienced significant improvements in CK, Hex4, and AST scores post-switch. The second-generation ERT avalglucosidase alfa (Nexviazyme) was designed to enhance cellular uptake via the conjugation of additional bis-mannose-6-phosphate residues (Dhillon, 2021). The study also revealed improvements in dyspnea, physical function, fatigue, and lower back pain. Avalglucosidase was well tolerated without infusion-associated reactions, and all patients on home infusions continued receiving ERT at home. Anti-drug antibodies were seen in 9/10 of patients on alglucosidase and 8/13 of those on avalglucosidase, with titers below 12,800 in a majority of patients. The study presents evidence that switching from alglucosidase to avalglucosidase may be associated with improved outcomes in certain patients with LOPD.

Gaucher disease (GD), the most prevalent lysosomal storage disorder, is a rare autosomal recessive disorder caused by genetic variations in *GBA* that encodes for the enzyme beta-glucocerebrosidase (GCase) that cleaves the beta-glucosidic linkage of glucosylceramide, an intermediate in glycolipid metabolism, leading to the toxic accumulation of sphingolipids in various organs (OMIM #230800). In a review by Mohamed and Al-Jasmi the potential of ABX as a pharmacological chaperone therapy for GD is highlighted, stressing the importance of addressing response variability in clinical studies to improve the management of this rare and complex disorder. Ambroxol (ABX) has emerged as a prospective enzyme enhancement therapy option, showing its potential to enhance mutated GCase activity and reduce glucosylceramide accumulation in GD-affected tissues of different *GBA* genotypes. The variability in response to ABX varies across different variants, highlighting the diversity in patients' therapeutic outcomes. Its oral availability and safety profile make it an attractive option, particularly for patients with neurological manifestations. Clinical trials are essential to explore further ABX's potential as a therapeutic medication for GD to encourage pharmaceutical companies' investment in its development.

In relation to APBD, the adult-onset form of glycogen storage disease type IV (GSD IV) (OMIM #232500), Gayed et al. present a case report involving the clinical course and diagnostic odyssey of a seven case series, including patients from the United States and Brazil. This disease is caused by biallelic pathogenic variants in *GBE1* which encodes the glycogen-branching enzyme (GBE) that adds branches to the growing glycogen molecule during the synthesis of glycogen, a storage form of glucose. Deficient GBE activity disrupts normal glycogen synthesis and leads to the formation of glycogen with elongated outer chains. Interestingly, the reported pathogenic variants involve a more recently identified deep intronic variant in *GBE1* as well as novel genotypes that expand our understanding of genotype-phenotype correlations. Also the results of a pilot application of patient-reported outcome measures to evaluate the domains of pain, fatigue, and quality of life are presented.

In regard to disorders affecting glucose-galactose metabolism, López-Mejía et al. present a case report that highlights the importance of Next-generation sequencing (NGS) panel in the diagnosis of the first Mexican female child reported with Congenital glucose-galactose malabsorption (CGGM; OMIM #606824), a rare autosomal recessive disorder that primarily causes chronic intractable diarrhea causing dehydration from the first day of life and that can quickly result in death. The patient presented with chronic diarrhea, anemia, jaundice, renal tubular acidosis, hyperammonemia, and initial hypertyrosinemia. A NGS panel for inborn errors of metabolism and congenital diarrhea, identified a homozygous variant in *SLC5A1* (c.1667T>C) that encodes the sodium-dependent glucose transporter-1 (SGLT-1), the primary transporter of monosaccharides in the small intestine and responsible for glucose and galactose active transport across the intestinal brush border. Introduction of galactose-glucose-free fructose-based diet led to a complete resolution of diarrhea and improved nutritional status. Also, in relation to galactose metabolism, Maroulis et al. present an original research article involving five cases of newborn patients affected by Galactosemia (OMIM #230400) identified through the National Newborn Screening program in Greece. Galactosemia is the most common and severe type of disorders related to inborn errors of galactose metabolism, caused by autosomal recessive mutations in the three genes that encode enzymes implicated in galactose catabolism (*GALT*, *GALK1*, and *GALE)* specifically in the conversion of α-D-galactose to glucose-1-phosphate. Patients were identified because of increased galactose levels. NGS identified eight rare nonsynonymous DNA variants of uncertain significance involving *GALT*, *GALK1* or *GALE*, four of them novel. Pathogenicity analysis through bioinformatic tools and *in silico* modeling as well as specific phenotypes for these cases are discussed. An additional contribution to the comprehension of galactosemia comes from a contribution by Panis et al. and members of the Galactosemia Network in a Mini Review article that presents the neurological impacts of classic galactosemia, detailing the ongoing challenges in understanding brain function associated with the disease. It discusses the role of abnormal glycosylation and myo-inositol deficiency, linking these biochemical dysfunctions to brain and neurodevelopmental issues. Treatment approaches, including dietary restrictions and emerging therapies, are examined, alongside their effectiveness in managing symptoms. Importantly, the review notes significant variability in neurological outcomes among individuals with classic galactosemia, highlighting that while some may experience worsening symptoms, many do not exhibit progressive cognitive decline.

In the more frequently studied aspect of carbohydrate metabolism Bawatneh et al. present an original research article presenting a study involving two Palestinian families affected by Diabetes Mellitus Type 1 (OMIM #222100). The use of WES allowed identification of variants, involving the *IGF1R* p.V579F variant, which follows autosomal dominant inheritance in one family and the *NEUROD1* p.P197H variant in the other family where it follows an autosomal recessive inheritance. This research marks a significant advance in understanding the genetic foundations of type 1 diabetes within this population. The study’s findings also underscore the importance of tailored genetic research in diverse populations to enhance disease understanding and management globally (Caliebe et al., 2022).

Overall, the articles in this Research Topic advance our understanding of the molecular, clinical, and therapeutic aspects of inborn errors of carbohydrate metabolism. They emphasize the importance of precision medicine, innovative diagnostics, and targeted therapies in addressing the significant challenges in diagnosis, management, and therapeutic intervention for these disorders (Might and Crouse, 2022; Trajanoska et al., 2023). It is our hope that the insights provided will inspire further research and foster collaboration to improve the lives of individuals and families affected by these metabolic diseases.
